# Molecular functions of nitric oxide and its potential applications in horticultural crops

**DOI:** 10.1038/s41438-021-00500-7

**Published:** 2021-04-01

**Authors:** Chengliang Sun, Yuxue Zhang, Lijuan Liu, Xiaoxia Liu, Baohai Li, Chongwei Jin, Xianyong Lin

**Affiliations:** 1 MOE Key Laboratory of Environment Remediation and Ecological Health, College of Environmental & Resource Sciences, Zhejiang University, 310058 Hangzhou, China; 2 Interdisciplinary Research Academy, Zhejiang Shuren University, 310015 Hangzhou, China; 3 Zhejiang Provincial Cultivated Land Quality and Fertilizer Administration Station, Hangzhou, China

**Keywords:** Plant signalling, Metabolism

## Abstract

Nitric oxide (NO) regulates plant growth, enhances nutrient uptake, and activates disease and stress tolerance mechanisms in most plants, making NO a potential tool for use in improving the yield and quality of horticultural crop species. Although the use of NO in horticulture is still in its infancy, research on NO in model plant species has provided an abundance of valuable information on horticultural crop species. Emerging evidence implies that the bioactivity of NO can occur through many potential mechanisms but occurs mainly through *S*-nitrosation, the covalent and reversible attachment of NO to cysteine thiol. In this context, NO signaling specifically affects crop development, immunity, and environmental interactions. Moreover, NO can act as a fumigant against a wide range of postharvest diseases and pests. However, for effective use of NO in horticulture, both understanding and exploring the biological significance and potential mechanisms of NO in horticultural crop species are critical. This review provides a picture of our current understanding of how NO is synthesized and transduced in plants, and particular attention is given to the significance of NO in breaking seed dormancy, balancing root growth and development, enhancing nutrient acquisition, mediating stress responses, and guaranteeing food safety for horticultural production.

## Introduction

Nitric oxide (NO) is a redox-active molecule that orchestrates a myriad of physiological and biochemical functions in biological organisms^1–3^. NO has been particularly well studied in mammals, where it regulates physiological processes of vital importance, such as neurotransmission and immunological and inflammatory responses^3–6^. Due to the active research and great achievements of NO in relation to human health issues, NO was named “Molecule of the Year” in 1992 by the journal *Science* and was the subject of the Nobel Prize for Physiology and Medicine in 1998. Despite the considerable amounts of attention this gaseous free radical has garnered within animal systems, the first identification of NO formation in biological systems occurred in plants^7^. Since then, the sources, signaling, molecular mechanisms, functions, and targets of NO have been thoroughly investigated in plants during the past few decades^1,8–12^.

**Fig. 1:**
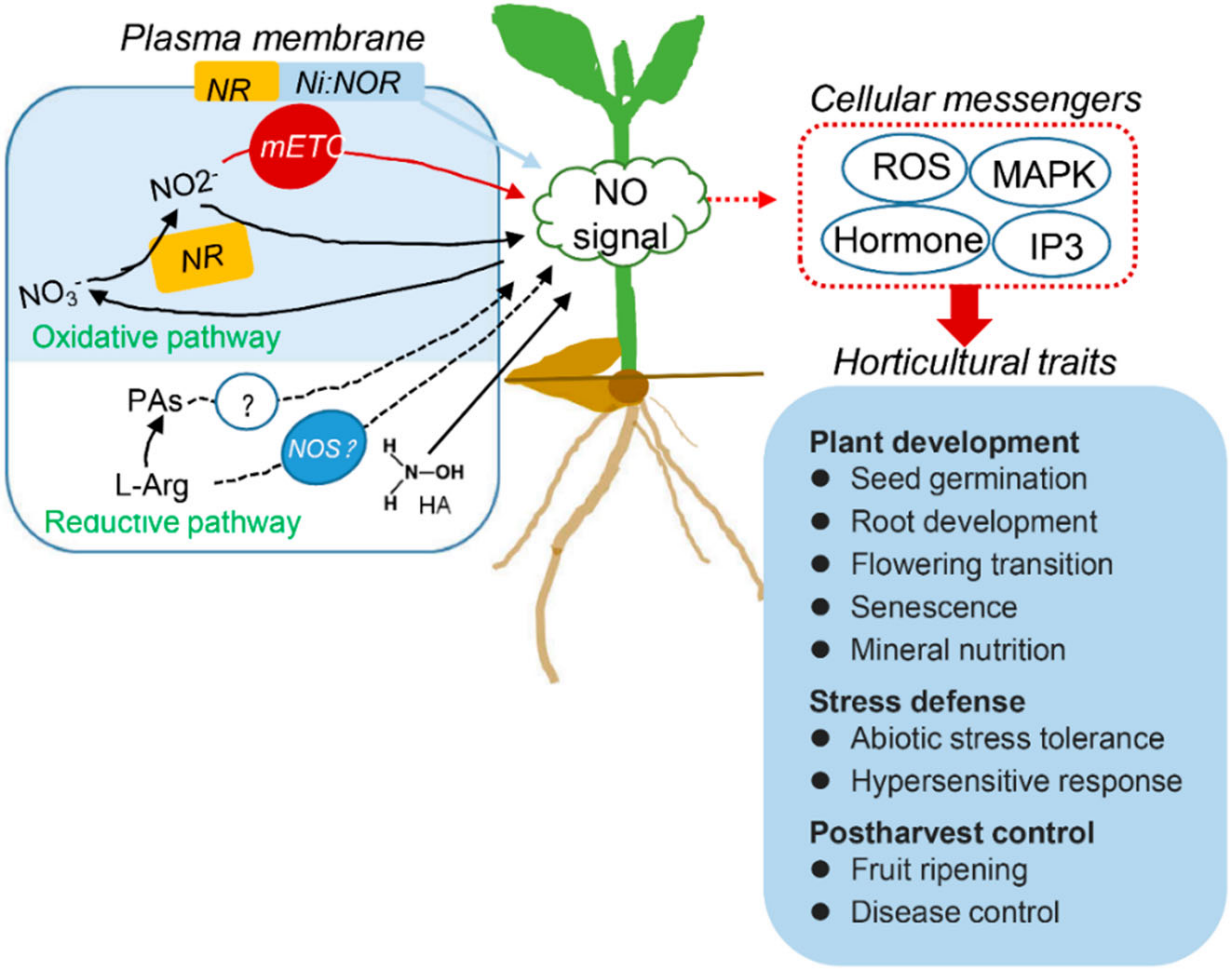
Sources of NO production and NO functions in regulating plant growth, development, and adaptive processes. The reductive pathway is based on the reduction of nitrite to NO, whereas the oxidative route relies on the oxidation of aminated molecules, such as l-Arg. The produced NO can be used to transduce external and internal signals to regulate plant development and stress responses by interacting with other cellular messengers. NR, nitrate reductase; Ni:NOR, NO-forming nitrite reductase; mETC, mitochondrial nitrite reduction; NOS, nitric oxide synthase; l-Arg, l-arginine; PA, polyamine; HA, hydroxylamine

The biological function of NO was first reported to be associated with plant immunity responses, initially in potato (*Solarium tuberosum*)^13^ and then in tobacco (*Nicotiana tabacum*)^14^, soybean (*Glycine max*)^15^, and Arabidopsis (*Arabidopsis thaliana*)^8^. These findings cemented NO as a crucial messenger in plant–pathogen interactions. Since then, many additional functions have been discovered (Fig. 1). NO regulates a variety of processes integral to plant growth and development, such as seed germination, root development, flower transition, and fruit ripening, as well as plant responses and adaptations to unfavorable environmental conditions^9,12,16–21^. Further, the complementary use of new tools and biological technologies have allowed the characterization of its biosynthesis routes and mode of action in plants.

Horticultural crops are an important component of agriculture for food as well as for nutritional security. The improvement in the growth, yield, and quality of horticultural crops has received a large amount of attention in recent years. Given the unique properties associated with plant growth and development, research on NO in horticultural crop species is substantially increasing^22–27^. The various roles of NO in plant biology make NO signaling a promising target for stimulating seed germination, optimizing root architecture, promoting plant growth and development, enhancing mineral nutrient acquisition, delaying postharvest fruit senescence, and increasing resistance to biotic and abiotic stresses. Moreover, with the advancement of genome-editing technologies, genetic manipulation of NO action in horticultural crops might have practical and promising prospects in the future.

## Current understanding of NO biosynthesis and bioactivity

### NO biosynthesis

Extensive research in different plant species and in various biological situations has revealed the coexistence of multiple routes with likely functions in plants, which depend on either reductive or oxidative mechanisms^9,11,28,29^. The reductive pathway is based on the reduction of nitrite to NO, whereas the oxidative route relies on the oxidation of aminated molecules (Fig. 1). However, significant controversy regarding our current understanding of NO biosynthesis in plant cells remains.

Reductive pathways are dependent upon nitrite as the primary substrate, which can be catalyzed by nitrate reductase (NR), NO-forming nitrite reductase (NOFNiR), and mitochondrial nitrite reduction^9,28,30,31^. Among these routes, NR-mediated NO generation from nitrite has been assumed to be the main enzymatic source in plants^32,33^. Since the early 2000s, a large number of independent studies have indicated a role for NR in the formation of NO integral to a variety of processes, including stomatal movement, the floral transition, auxin-regulated lateral root formation, root hair development, and various stress defense responses^18,34–37^. It should be noted that NR primarily catalyzes the reduction of nitrate to nitrite, and nitrite reduction constitutes only 1% of NR activity^30^, indicating extremely low levels of NO production resulting from this route under normal conditions. However, under acidic conditions or under high concentrations of nitrite and low nitrate, NR-dependent NO emission is accelerated in plant cells. Subsequent work showed that posttranslational regulation of NR via the phosphorylation of a conserved serine residue enables NR to bind to 14-3-3 proteins, leading to NR inactivation and degradation^38^. In addition to NR, the participation of a plasma membrane-bound nitrite reductase (Ni:NOR) in the germination of NO was first reported in tobacco^39^, with activity being limited in the roots. In conjunction with an apoplastic membrane-bound NR, Ni:NOR produces NO in the apoplast and plays pivotal roles in sensing nitrate availability and regulating mycorrhizal colonization^40,41^. Although NOFNiR has been suggested to be a component of another NO-producing route from nitrites, the functionality of this system has been detected only in *Chlamydomonas reinhardtii*^42^ and remains largely unknown in higher plants. Like NR and NOFNiR, other enzymes such as xanthine oxidase containing a molybdenum cofactor in their structure show the potential to produce NO from nitrite in plants^43^. At present, however, no information is available on their NO synthesis ability in plants. Other proposed reductive routes for NO production include the mitochondrial electron transport chain (mETC), as mETC inhibitors prevent NO biosynthesis in algae and tobacco^44,45^.

Regarding the oxidative pathway contributing to NO production, several lines of evidence demonstrate that plants are able to synthesize NO by oxidizing N-containing molecules, similar to the dominant pathway in animals. The oxidation of arginine to citrulline and NO, which is catalyzed by three distinct nitric oxide synthases (NOSs) in mammals, has also been proposed to occur in higher plants^46,47^. Several studies have sought to identify and characterize NOS homologs in the plant kingdom. A candidate, initially named AtNOS1, was identified in *Arabidopsis* based on its similarity with a protein involved in NO generation in the snail *Helix pomatia*^48^. However, researchers subsequently characterized it as a functional small GTPase and therefore renamed nitric oxide associated 1 (AtNOA1)^49^. In the beginning of 2010, a related protein with 45% sequence similarity to the human eNOS sequence was identified in the green alga *Ostreococcus tauri*^50^. However, a systematic search for homologous human nNOS sequences in more than 1000 land plants and algae showed that 15 typically identified NOSs belonged to algal species, whereas no NOS homologs were found in the genomes of land plants^51^. These findings and previously unsuccessful attempts to identify candidates suggest that canonical NOSs might not exist in higher plants. According to phylogenic relationships, NOS was later lost in land plants. However, it cannot be excluded that some key motifs or single residues important for NOS activity are conserved; thus, animal NOS inhibitors also function in plants. It has been proposed that hydroxylamine-mediated NO synthesis constitutes another potential oxidative route^52^, and NO produced in this way is considered to participate in regulating seed germination and tolerance to abiotic stresses, such as salt, oxidative and drought stresses. However, the subcellular location and level of hydroxylamine-mediated NO currently are unknown. Several works have reported that increased levels of polyamines such as spermine and spermidine resulted in NO release in several plant species^53^. Although the underlying mechanism has not yet been resolved, polyamine-induced NO has been suggested to regulate root development and embryogenesis as well as plant responses to cadmium and drought stress^54^.

### Transfer of NO bioactivity

**Fig. 2:**
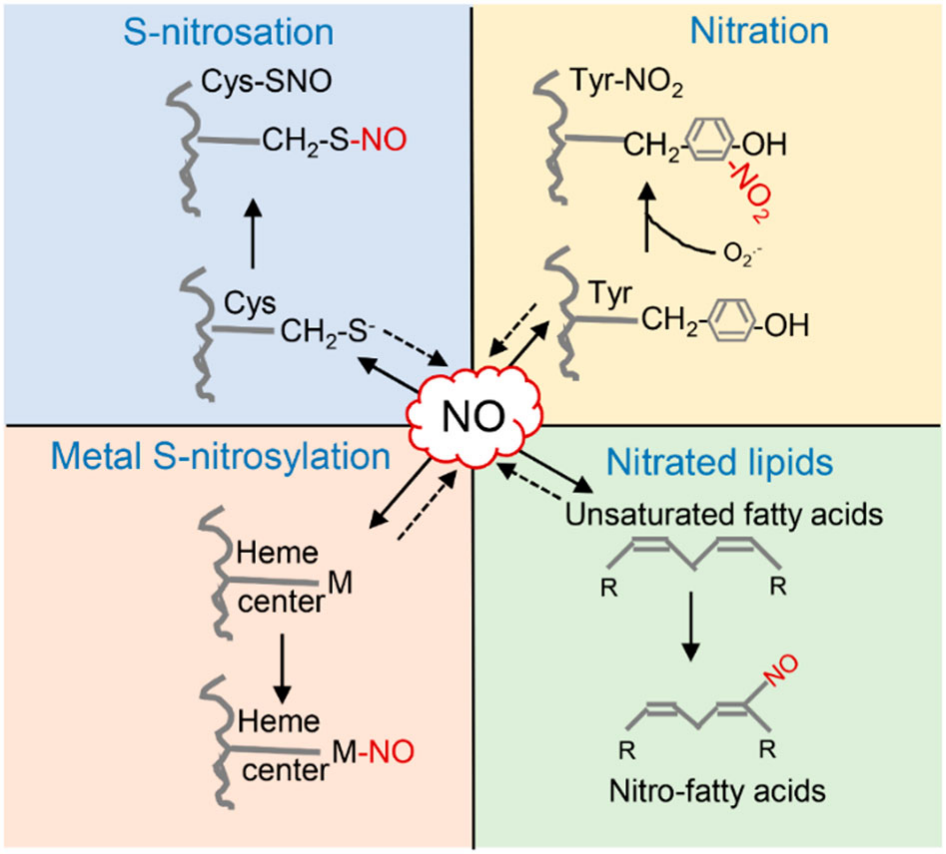
NO-dependent posttranslational modifications in plants. *S*-nitrosation, the covalent and reversible attachment of NO to a reactive thiol group of cysteine forming an *S*-nitrosothiol. Metal *S*-nitrosylation, in which an NO radical donates electrons and therefore reacts with transition metals. Tyrosine nitration is mediated by ONOO^−^, an NO-derived species, resulting in the formation of a 3-nitrotyrosine residue. Nitration of unsaturated fatty acids forms nitro-fatty acids. All these processes except nitration are assumed to be reversible in plants. The dotted arrows indicate that all the products generated can release NO in plant cells

The molecular details underpinning exactly how NO as a signaling messenger is translated into biological function have been under extensive research in recent decades. In animals, NOS-mediated NO is perceived by soluble guanylate cyclase through binding to prosthetic hemes, leading to the production of cyclic 3′,5′-guanosine monophosphate (cGMP)^2,55^. Although low levels of cGMP have been detected in plants, a NO-cGMP signaling pathway, however, might not exist in plants according to recent bioinformatic analyses in over 1000 plant species^51^. In the absence of an NO receptor, NO likely conveys its bioactivity through chemical interactions with specific residues of target proteins that undergo NO-dependent posttranslational modifications (PTMs) (Fig. 2)^9,28,29^.

Among these PTMs, *S*-nitrosation is key, which describes the covalent and reversible attachment of NO to a reactive thiol group of cysteine forming an *S*-nitosothiol^56,57^. Using biotin switch approaches, Lindermayr and coworkers first identified *S*-nitrosated proteins in *Arabidopsis* leaf and cell suspension protein extracts after exogenous NO application^58^. These proteins participate in various biological activities, such as metabolism, cytoskeleton, cellular signaling, redox homeostasis, and stress responses. That same year, an *Arabidopsis* gene with sequence similarity to that of *S*-nitroglutathione reductase (GSNOR), which regulates the formation and turnover of *S*-nitrosothiols (SNOs) in plants, was identified, and it was also found that *AtGSNOR1* controls the extent of global *S*-nitrosylation in *Arabidopsis*^59^. Moreover, it has been reported that NO curbs the extent of cell death development during the hypersensitive response (HR) by *S*-nitrosation of NADPH oxidase, and Cys 890 was identified as the *S*-nitrosation site, thus inhibiting reactive oxygen species (ROS) production^60^. Several reports have pointed out that NO-triggered *S*-nitrosation of enzymes and proteins participating in the transport and signal transduction of distinct hormones exerts NO regulatory actions during plant development and defense responses^9,61^. For example, NO directly influences auxin perception and signaling by triggering the *S*-nitrosation of TRANSPORT INHIBITOR RESPONSE 1 (TIR1), an auxin receptor protein, at distinct (Cys 140 and Cys 480) residues^62^. This PTM of TIR1 promotes its interaction with AUXIN/INDOLE-3-ACETIC ACID (AUX/IAA) proteins, which repress the transcription of auxin-responsive reporter genes, facilitating AUX/IAA degradation and auxin signal transduction. By promoting the *S*-nitrosation and degradation of positive regulators of abscisic acid (ABA), such as open stomata 1/sucrose nonfermenting 1-related protein kinase 2–6 and ABA-insensitive 5, NO negatively regulates ABA signaling^63,64^. It has also been reported that NO negatively regulates cytokinin signaling by inhibiting phosphorylation activity via the *S*-nitrosation of Cys 115 of HISTIDINE PHOSPHOTRANSFER PROTEIN 1 (AHP1)^65^. To date, there is no doubt that this redox-based *S*-nitrosation constitutes an important strategy to convey NO signals into biological functions. A fundamentally critical question is how the signaling of NO as a redox signaling molecule via *S*-nitrosation is deactivated or downregulated. A growing body of evidence has revealed that GSNOR, an evolutionarily conserved NADH‐dependent reductase, controls GSNO contents and subsequently global *S*-nitrosation levels, and this process is an important regulatory feature of NO bioactivity^59^. Currently, emerging evidence suggests that GSNOR-mediated denitrosylation has crucial roles in plant growth and development, disease tolerance, iron toxicity, thermotolerance, hypoxic responses, and salinity tolerance^66–70^, which are discussed in more detail later in horticultural crop species.

NO can also form complexes with the heme center of metalloproteins through metal *S*-nitrosylation reactions, impacting their activities^29^. Most studies have shown that hemoglobin, lipoxygenase, catalase, cytochrome c-oxidase, and ascorbate peroxidase are putative *S*-nitrosylation targets in plants^32,71^. Another important NO-mediated PTM is tyrosine nitration, leading to the generation of 3-nitrotyrosine, which is associated with nitro-oxidative damage in biological systems^72^. Tyrosine nitration has been extensively investigated in plants under adverse conditions. For example, nitroproteome analysis revealed an accumulation of tyrosine-nitrated proteins in sunflower seedlings exposed to high temperature^73^, leaves of *Citrus* plants under salinity stress^74^, and *Arabidopsis* plants under arsenic stress^75^. Generally, nitration improves the possibility of the protein being degraded by the proteasome^76^. Based on the above, the possible involvement of proteasomal degradation in conveying NO signals via tyrosine nitration needs to be better understood in plants.

More recently, nitration of unsaturated fatty acids, forming nitro-fatty acids, by NO-related species has been identified in plants^77–79^. The initial discovery of nitro-fatty acids in olive oils and olives promoted the identification and characterization of this molecule in plant species such as *Arabidopsis*, pea (*Pisum sativum*), and rice (*Oryza sativa*)^77,79^. A few studies have reported that the accumulation of nitro-fatty acids such as nitro-linolenic acid and nitro-oleic acid were significantly induced by stresses^79,80^, and these molecules are probably novel key mediators in the defense mechanism mediated by NO in response to different biotic and abiotic stress situations.

Extensive research has established that NO pervades almost all aspects of plant development and the response to particular environmental cues^16–21,81^. A current central question is how NO, a single redox-active molecule, encodes complex information with particular specificity. Emerging evidence suggests that a multilayered molecular framework governs NO signaling specificity in a particular cellular microenvironment^82^. Predominantly, the temporal and spatial nature of different NO-based PTMs in discrete protein functions confers specificity to NO-based signaling^57,60,82^. It is well established that cysteine thiols experience a redox continuum of alterations generating redox-related functional groups, including SNO, sulfenic acid (S–OH), disulfide (S–S), *S*-glutathionylation (S-SG), sulfinic acid (S–O_2_H), and sulfonic acid formation (S-O_3_H)^83^. It turns out that each modification leads to different conformational changes and, consequently, distinct cellular outcomes^84^. Another well-established strategy in mammals for maintaining specificity in NO signaling is the proximity of NO to the target cystein^82^; however, this proximity-based mechanism has not been demonstrated in plants. Similar to other PTMs, the decoration of cysteine residues with NO is thought to be reversible, except for sulfonic acid (S-O_3_H)^82,83^. Increasing evidence has demonstrated that reversal of the binding of cysteine residues with NO, rather than their formation, constitutes an important strategy to convey specificity to NO-related redox signaling^66,85^. In this context, GSNOR regulates GSNO levels, and thioredoxin h5 (TRX h5), acting as a selective protein-SNO reductase, has emerged as a key mechanism for denitrosylation^57,66,70,85^. *Arabidopsis* seedlings with impaired *GSNOR1* function exhibited increased levels of protein-SNO and deficiencies in development, immunity, and thermotolerance^59,70,86^. Emerging genetic and biochemical evidence indicates that plant TRX h5 discriminates between protein-SNO substrates, achieving specific, reversible protein-SNO signaling in plant immunity^85^. Emerging evidence has shown that another major route for transferring NO bioactivity is through impinging other ubiquitous and fundamental PTMs, such as SUMOylation, phosphorylation, persulfidation, and acetylation^87^. Through *S*-nitrosation, NO is able to modulate these PTMs, tailoring the cellular response to various stimuli. Finally, distinct differences between ROS and RNS can also establish specificity of redox signaling outputs^88,89^.

### NO regulates the development of horticultural crops

Extensive knowledge on the multiple effects of NO on regulating plant development processes is available in model plant species such as *Arabidopsis* and economically important crop species, such as rice and wheat. Emerging evidence illustrates the value of NO in providing important horticultural traits (Fig. 1). Increased seed germination percentage and seedling growth, as well as biomass accumulation and yield in a number of vegetables, flowers, and fleshly fruits, have been reported after treatment with NO-releasing compounds (Table 1)^10,90–92^. Furthermore, the recent identification of the first GSNOR enzyme in higher plants has opened the door to studying NO metabolism in plant growth and development under physiological and stressful conditions using a genetic approach. In *Arabidopsis*, it was found that *AtGSNOR1* controls several pivotal processes of plant development^86,93^. For example, loss-of-function mutants in *GSNOR1* in *Arabidopsis* resulted in a loss of apical dominance and reduced hypocotyl elongation^86,93^. Recently, multiple *GSNOR*-mediated developmental processes that regulate *S*-nitrosation have also been reported in tomato (*Lycopersicon esculentum*) seedlings. Knockdown of GSNOR resulted in an increased germination rate, severe inhibition of root and hypocotyl growth, substantially decreased photosynthesis, changes in leaf shape, and, important, reduced fruit yield^94,95^.

**Table 1:** Summary of NO in the growth and development processes of horticultural crop species

Development stage		Plant species	NO function	Reference
	Dormancy and germination	*Lactuca sativa*	Promotes seed germination and de-etiolation and inhibits hypocotyl and internode elongation	Beligni and Lamattina, 2000
		*Lycopersicon esculentum*	Stimulates seed germination	Piterková et al., 2012
		*Cucumis sativus*	Accelerates seed germination and increases budding seed weight	Fan et al., 2013
		*Malus domestica*	Breaks embryo dormancy by stimulating ethylene synthesis	Gniazdowska et al., 2007; Krasuska et al., 2012; 2014; 2017; Gniazdowska et al., 2010
		*Coriandrum sativum*	Simulates germination and seedling growth	Ji et al., 2015; Panngom et al., 2018
		*Solanum lycopersicum*	Stimulates seed germination	Piterkova et al., 2012
		*Amaranthus retroflexus*	Markedly releases the seed dormancy	Liu et al., 2011
		*Brassica juncea*	Advances seed germination under copper stress	Rather et al., 2020
	Adventitious root (AR)	*Cucumis sativus*	Stimulates adventitious root formation by cooperating with cGMP, MAPK, and PA signals	Pagnussat et al., 2002; 2003; 2004; Lanteri et al., 2006; 2008; Qi et al., 2017; Xuan et al., 2012; Zhu et al., 2016
			Protect roots against oxidative stress induced by salt stress	Shi et al., 2007
	Lateral root (LR)	*Lycopersicon esculentum*	Enhances LR development and modulates the expression of cell cycle regulatory genes during LR formation	Correa-Aragunde et al., 2004; 2006
			Affects cellulose content	Correa-Aragunde et al., 2008
			Involvements in *A. brasilense*-induced LR formation	Creus et al., 2005
			Enhances LR development under CO and CO_2_	Guo et al., 2008; Wang et al., 2013
	Root hair (RH)	*Lactuca sativa*	Functions as a positive regulator of RH development	Lombardo et al., 2006
	Reproductive growth	*Litchi chinensis*	Promotes reproductive growth, promote abscission of rudimentary leaves, encourages panicle development, and promotes the expression of the flowering-related genes	Liu et al., 2015; Zhou et al., 2012
		*Cucumis sativus*	Involvement in pollen germination and pollination	Sírová et al., 2011
		*Lilium longiflorum*	Involvement in growth regulation and reorientation of pollen tubes	Prado et al., 2004
		*Paulownia tomentosa*	Involvement in UV-inhibited pollen germination and tube growth	He et al., 2007
		*Pinus*	Modulates cell wall construction in pollen tubes	Wang et al., 2009
		*Olea europaea*	Involvement in both the papillae and exudates on the stigma surface	Zafra et al., 2010
	Fruit ripening	*Actinidia chinensis*	Decreases ethylene production and extends postharvest life	Zhu et al., 2010
		*Prunus salicina*	Delays ripening and alleviates chilling injury during cold storage	Singh et al., 2009
		*Mangifera indica*	Increases fruit firmness, reduces softening, and delays fruit color development and ripening	Zaharah and Singh, 2011; Hu et al., 2014; Ruan et al., 2015
		*Prunus persica*	Maintains firmness and lowers ethylene production	Saba and Moradi, 2017; Han et al., 2018
		*Fragaria ananassa*	Reduces ethylene production and extends postharvest life	Leshem and Pinchasov, 2000
		*Capsicum annuum*	Delays fruit ripening	Chaki et al., 2015

### NO breaks seed dormancy and germination of horticultural crops

Seed germination is tightly regulated by a combination of external conditions and endogenous signals, maximizing growth and crop yield. For a long time, various nitrogenous compounds such as nitrate and nitrite have been used to break dormancy and stimulate seed germination in agricultural production^96,97^. Subsequent data have revealed that the promotion of dormant seed germination by these nitrogenous molecules probably occurs through NO generation. In initial experiments with lettuce (*Lactuca sativa*) seeds, NO stimulated seed germination in light-dependent situations^91^. In tomato seeds, NO scavengers maintained seed dormancy and counteracted the stimulating effect of fluridone^98^. It is well known that smoke is commonly used in horticulture to break dormancy and promote seed germination of some vegetables, such as lettuce and celery^99^. It is now clear that NO is among the essential active compounds responsible for seed germination stimulated by smoke. A seed of many edible fruit tree species such as apple (*Malus domestica*) and pomegranate (*Punica granatum*) shows the most pronounced and complex forms of dormancy, which is completely removed by several months of cold or warm stratification. Pretreatment with an NO donor (5 mM Sodium nitroprusside (SNP)) promoted embryo germination by nearly 60% after 8 days, whereas it promoted germination by only 16% in nontreated seeds, and this effect was associated with the modulation of ROS metabolism by NO in the embryos at the early germination stage^100^. When seed dormancy is released, a large amount of NO accumulates in the axes of embryos of apple seeds and in the endosperm of *Sechium deule*^100,101^. Seed dissection suggests that the aleurone layer perceives and responds to NO during seed embryogenesis. Moreover, NO is able to protect the seed germination of various horticulture species under stress conditions, and presoaking attenuated the inhibition of seed germination and early growth of cucumber (*Cucumis sativus*) and pak choi (*Brassica chinensis*) under salt stress^90,102^.

**Fig. 3:**
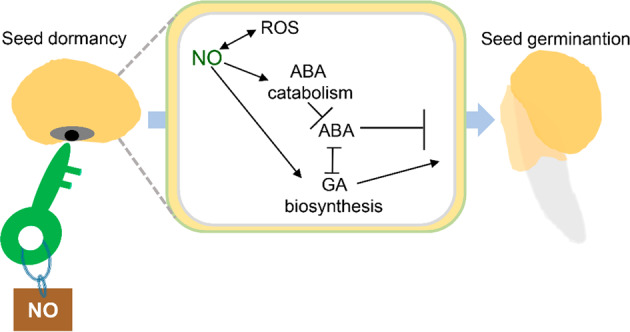
Functional interactions among NO, ABA, and GAs during seed germination. NO induces ABA catabolism and promotes GA biosynthesis, leading to dormancy release and germination. The arrows and bars indicate positive and inhibitory effects, respectively. ABA, abscisic acid; GA, gibberellic acid; ROS, reactive oxygen specie

A variety of phytohormones have been described to regulate the seed-to-seedling transition. It is well known that seed dormancy is strictly dependent on ABA concentration, whereas seed germination requires enhancement of gibberellic acid (GA) synthesis and signaling. During seed dormancy release, extensive cross-talk between NO and these two phytohormones has been observed^61,103^. For example, endogenous NO production increased after ABA treatment, which was proposed to ameliorate the repressive effect of ABA, and NO accumulation generally decreases ABA contents in seeds^104^. Intensive amounts of research have been dedicated to unraveling the mechanisms underlying the interactions among the NO, ABA, and GA signaling networks during seed germination (Fig. 3), leading to the identification of several NO targets. Recently, evidence has revealed that NO enhances the degradation of ABSCISIC ACID INSENSITIVE 5 (ABI5) via *S*-nitrosation or promotes the degradation of group VIII ETHYLENE RESPONSE FACTORS (VIII ERFs) through the *N*-end rule pathway, resulting in seed germination in *Arabidopsis*^64^. Although still far from completely known, the mechanistic basis of NO-mediated seed dormancy release occurs presumably through synthesis and perception of NO in the aleurone layer, which in turn leads to ABA catabolism and GA biosynthesis in the embryo^10^. The resulting GA promotes cell wall loosening of *EXPANSIN* (*EXPA*) expression, facilitating the degradation of the physical barrier and allowing root emergence and germination^105^. It should be noted that during the regulation of certain biological processes, such as stomatal closure and antioxidant defense responses, NO acts downstream of ABA^106^. These results indicate a certain level of specificity in NO–ABA interaction mechanisms, which might depend on physiological events or the type of plant species, tissue or organ considered.

In addition to promoting seed germination, a recent study showed that NO also plays an important role in seed oil content and fatty acid composition^107^. Compared with the Arabidopsis wild type, the *Arabidopsis* mutant *gsnor1* produced smaller seeds and reduced oil content. Moreover, the fatty acid composition was significantly altered in *GSNOR1* RNAi2 transgenic lines, which showed lower GSNOR activity compared with that of the wild type, with increases in palmitic acid (C16:0), linoleic acid (C18:2), and linolenic acid (C18:3) and significant decreases in stearic acid (C18:0), oleic acid (C18:1), and arachidonic acid (C20:1)^107^. This study provides a potential approach to improve the seed oil content by regulating NO signaling in siliques of oil crop species.

### NO balances root growth and development of horticultural plants

The root architecture system involves the coordinated growth of primary roots, lateral roots, and adventitious roots, which is tightly controlled by multiple genetic and environmental factors. In recent decades, a large number of experiments have highlighted the role of NO as a central regulator in auxin-orchestrated root growth and the development of plant roots^9,24,108,109^. Detailed information concerning this process was provided by a series of studies conducted by Lamattina and coworkers with cucumber and tomato plants^24,92,108,110^.

The linkage between NO and auxin was first reported in cucumber during adventitious root formation in 2002 (ref. ^108^). The effects of NO application mimicked those of IAA, which stimulated adventitious root development in cucumber hypocotyls, whereas the NO scavenger completely blocked IAA-promoted adventitious root growth. A series of studies demonstrated that a number of secondary messengers participate in NO-mediated adventitious root growth. It has been observed that phosphatidic acid (PA) derived from both phospholipid signaling and the MAPK cascade acts as an agonist and probably works downstream of NO and auxin during this process^92^.

Lateral roots are a major determinant of root architecture, which is predominantly associated with auxin activation. Recently, NO has been considered to be a crucial downstream messenger in auxin signaling, promoting lateral root formation^9^. The first evidence on the stimulatory effect of NO on lateral root development was identified in a horticultural crop species. It was found that in tomato seedlings, the NO donor was able to induce lateral root emergence and elongation while inhibiting primary root growth in the same way that auxin does^24^. However, depletion of NO led to complete abolition of lateral root formation and partly restored primary root elongation. According to Correa-Aragunde et al.^111^, NO regulates the expression of genes involved in the cell cycle in tomato pericycle cells, which in turn results in lateral root formation. Under metal stress, it has been observed that NO is able to mitigate the inhibition of lateral root development caused by toxic soil pollutants such as cadmium and arsenic^112^. It was proposed that NO is linked to ROS, which cause auxin oxidation, thus maintaining auxin homeostasis and favoring lateral root formation^112^. Recent studies have also demonstrated that NO plays a critical role in controlling lateral root development triggered by other environmental stimuli and regulators, such as plant growth-promoting rhizobacteria^113^, carbon monoxide^114^, and elevated carbon dioxide^115^, in tomato and other horticultural crop species.

From the above effects of NO, it was inevitable that potential effects of NO in modulating the development of root hairs, which are derived from certain epidermal cells termed trichoblasts, be proposed. Not surprisingly, in 2006, NO was shown to promote the differentiation of trichoblasts in developing root hairs in lettuce plants^116^. Increased NO contents in the root hairs after 1-naphthyl acetic acid (NAA) treatment probably suggested that auxin-induced root hair formation is NO dependent. Current research suggests that NO appears to regulate root hair formation by acting on vesicle formation and trafficking, as NO synthesis mutants demonstrated altered vesicle trafficking and decreased root hair length. More recently, Lombardo and Lamattina reported that NO modulates root hair growth by acting on cytoskeletal organization by cooperating with ABA^117^.

Conversely, it has been reported that NO is able to inhibit root growth. Exogenous application of NO donors in tomato reduced overall primary root growth^24^. However, knowledge of the molecular mechanisms through which NO inhibits root growth is still limited. Using phenotypic, cellular, and genetic analyses in *Arabidopsis*, Fernández-Marcos et al. demonstrated that the disruption in auxin transport and response via a PIN1-dependent mechanism to high NO led to a reduction in root meristem activity^118^. This mechanism also underlies cadmium-induced inhibition of primary root growth through NO accumulation^119^. A direct influence of NO on auxin perception and signaling has been demonstrated based on the observation that TIR1, an auxin receptor protein, underwent *S*-nitrosation, enhancing TIR1–AUX/IAA (transcriptional repressors) interaction, facilitating AUX/IAA degradation, and subsequently promoting gene expression^62^. However, with respect to much of the progress concerning the understanding of the molecular mechanisms in *Arabidopsis*, whether these findings are of physiological relevance in horticultural crop species needs be thoroughly investigated.

## NO function in plant nutrition and abiotic stress in horticulture

Horticultural production presents a significant challenge for nutrient management because many crops require large quantities of fertilizer to maximize yields and profits. Increased NO levels have been observed in different plant tissues following changes in nutrient supplies^26^. Given the significant impact of NO on root system architecture, it is not surprising that NO plays essential roles in plants experiencing mineral nutrient imbalances.

To our knowledge, the first evidence indicating a direct modulation of plant mineral nutrition by NO originated from the ability of NO to cope with iron (Fe) deficiency symptoms in maize (*Zea mays*)^120^. Since then, extensive information supporting the pivotal role of NO in Fe nutrition, metabolism, transport, and availability in monocot and dicot plants has been accumulated, including various horticultural crop species^121^. In tomato, Fe deficiency causes rapid NO production in the root epidermis^25,122^. The resulting NO positively regulates the expression of genes associated with Fe uptake, including *LeFER*, *LeFRO1*, and *LeIRT1*. Similarly, the enhancing effect of NO on the expression of Fe-acquisition genes has been observed in cucumber seedlings under Fe deficiency conditions^123^. Thus, exogenous NO could alleviate the inhibition of photosynthesis and growth in Chinese cabbage (*B. chinensis*) cultivated under Fe deficiency^124^. These NO-mediated Fe deficiency responses occurred not only in herbaceous plant species such as *Arabidopsis*, tomato, peanut, and cucumber but also in fruit tree species grown in calcareous soil. For example, in the woody plant species *Malus xiaojinensis*, Fe deficiency stimulated marked accumulation of NO in the root elongation zone, whereas eliminating NO arrested root hair formation, blocked ferric chelate reductase activity, and prevented the upregulated expression of critical Fe-related genes^125^. Moreover, NO has been described to improve Fe utilization efficiency. In *Arabidopsis*, putrescine-induced NO could alter the cell wall composition, leading to the mobilization of Fe from roots to shoots and increases in available Fe levels^126^. These results are in contrast to those reported by Ye et al. on tomato plants, who suggested that elevation of NO might be an unfavorable factor under Fe deficiency because it could lead to Fe immobilization in the root apoplast^127^. It is possible that the discrepancy between these studies might be attributed to different plant species and treatment methods.

NO is emerging as a crucial player controlling the uptake and homeostasis of most macronutrients, including nitrogen (N), phosphorus (P), and potassium (K). These elements are key components of many macromolecules, such as nucleotides, amino acids, and proteins. Nitrate (NO_3_^−^), one of the most abundant sources of N in agricultural and horticultural systems, and NO are metabolically connected via NR, a key enzyme involved in both nitrogen acquisition and NO generation^32,128^. Increased NO_3_^−^ supplies rapidly caused NO generation in the first few minutes, and NO, in turn, participates in NO_3_^−^-mediated root architecture alterations, thus exhibiting involvement in N perception and uptake^129–131^. On the other hand, NO regulates NR activity in the Chinese cabbage pak choi (*B. chinensis*) and tomato^132,133^, depending on the levels of NO_3_^−^ supply, and the regulatory effects of NO on NR activity probably operate at the posttranslational level. This proposal was investigated later in *Arabidopsis*. More recently, biochemical and genetic approaches proved that NO is at the center of fine tuning nitrogen homeostasis in plants, as NO derived from nitrate assimilation can suppress both nitrate uptake and reduction by transporters and reductases, which in turn control its own generation^128^. Importantly, NO plays multiple roles in nitrogen-fixing symbiosis, which is performed mainly by legumes to enhance nitrogen acquisition. The sources and effects of NO have been extensively reviewed during root nodule symbiosis^134,135^. Under P and K nutrient deficiency, NO is involved in remodeling the root system and increasing the transport activity of these two elements^26^. In a study in white lupin (*Lupinus albus*), NO was shown to be involved in P deficiency-induced cluster root formation and citrate exudation^136^. It has also been reported that NO acts upstream of ethylene in cell wall P reutilization in rice plants under P deficiency^137^. After K restriction, NO significantly increased in plant cells^138,139^. The induced NO negatively regulates the Shaker-like K^+^ channel (AKT1), indicating the role of NO in K homeostasis movement at the cellular level^139^. This phenomenon might be particularly relevant when plants are grown under stress conditions, such as drought and heat stress.

There are relatively few studies considering the relationship between NO and deficiencies in other nutrients in plants, such as copper (Cu), zinc (Zn), manganese (Mg), and boron (B), which are required in low amounts but are toxic at high levels^26,140,141^. The most studied interactions between NO and micronutrients describe the beneficial behavior of NO under excessive amounts of Cu. In periwinkle (*Catharanthus roseus*), a medicinal plant species, NO alleviates Cu toxicity by enhancing the activity of ATPase and stimulating the accumulation of secondary metabolites^142^. Similar results have also been observed in tomato seedlings under Cu stress^143^. Farag et al. reported that NO protected watermelon seedlings from B-induced injury by reducing B accumulation and preventing oxidative damage^141^. However, micronutrient deficiencies are common in horticulture due to intensified agricultural practices and unbalanced fertilizer applications; Zn deficiency is the most widespread, followed by B, Mg, and Cu deficiencies^144^. In *Arabidopsis*, it has been reported that NO regulates Mg deficiency‐induced root hair morphogenesis^145^. However, there is a lack of knowledge about NO participation in horticultural crops exposed to micronutrient deficiencies.

**Fig. 4:**
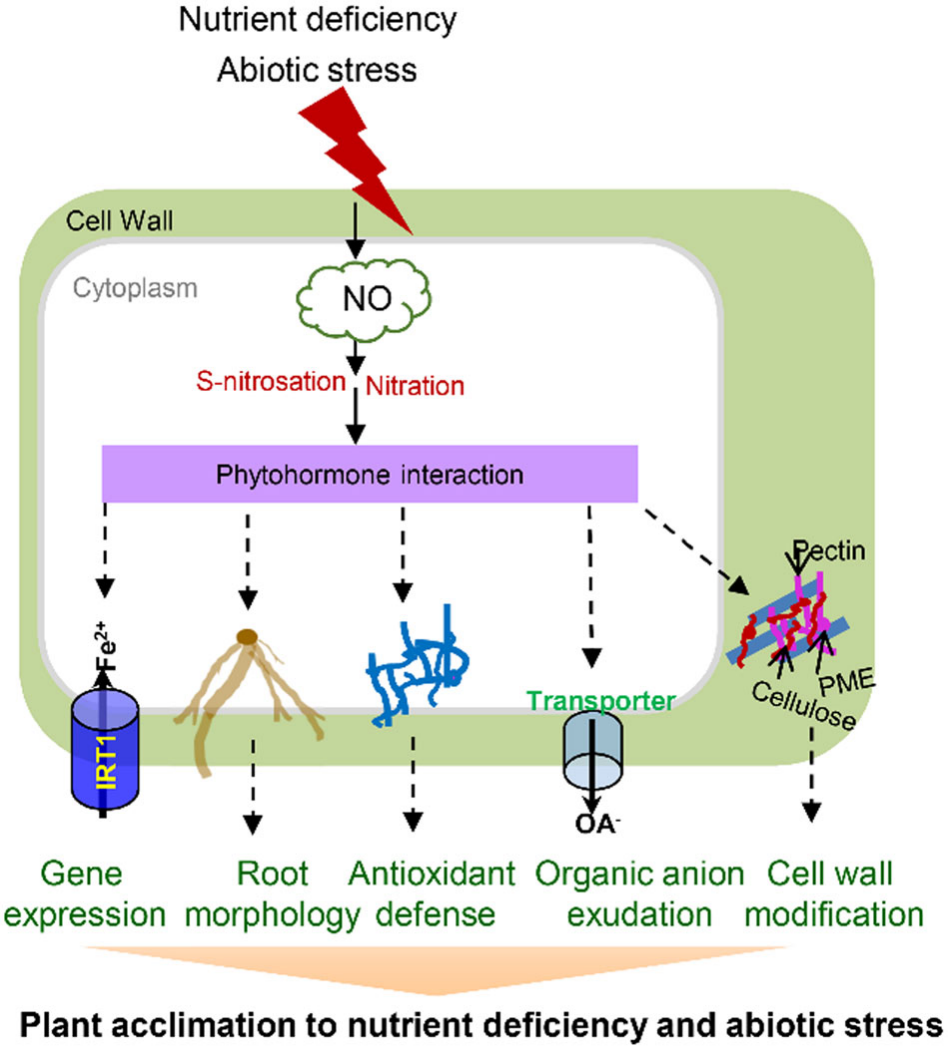
Generalized mechanisms involving NO under nutritional and abiotic stress. Adverse environments rapidly induce the accumulation of NO, which elicits nutrient acquisition and stress defense responses, including enhanced defense gene expression, altered root morphology, improved antioxidant defense, accelerated organic acid exudation, and changed cell wall composition. Arrows indicate positive effects

In addition to nutrient deficiency, abiotic stresses such as salinity, drought, and extreme temperature are the main constraints drastically limiting horticultural crop productivity worldwide. Increasing amounts of data have indicated a crucial role for NO in a number of stress responses not only for model plant and major cereal crop species but also for horticultural crop species^21,146^. NO was recently proposed to mediate the plant response to salt stress on the basis of its toxicity and signaling functions. Under salt stress, NO has been found to improve seed germination and plant growth of pak choi (*B. chinensis*), sunflower (*Helianthus annuus*), and cucumber by alleviating oxidative stress or regulating secondary metabolism^90,147,148^. In recent years, attention has been given to the NO-dependent PTM of proteins in response to salt stress. In olive leaves, salt stress induces a large amount of NO production and an increase in tyrosine-nitrated proteins, consequently leading to nitrosative stress in plant cells^149^. Later, in sunflower seedlings, David et al. suggested that rapid NO accumulation and protein tyrosine nitration provided longevity to oil bodies for plant survival under salt stress^150^. Pretreatment of citrus (*Citrus aurantium*) plants sensitive to salinity with an NO donor enhanced the capacity of these plants to withstand high salinity. Further proteomic analysis suggested that protein *S*-nitrosation appears to mediate the acclimation of citrus seedlings to salt stress^151^. Compared with salt stress, drought stress is even more pervasive and damaging. In addition to model plant species, increasing evidence has revealed a function for NO in mitigating drought stress in horticultural production. For example, NO protected the ultrastructure of mesophyll cells and promoted adventitious root formation in marigold (*Tagetes erecta*) under drought stress^152^. Using two sugarcane genotypes (*Saccharum* spp.) with different drought tolerances, Silveira et al. found that NO metabolism is more active in the roots of drought-tolerant genotypes than in those of drought-sensitive genotypes^153^. In this context, NO might stimulate root formation and enhance water uptake in the tolerant genotypes. Improved drought tolerance by NO in some model plant species is associated with its ability to enhance antioxidant system, proline, and osmolyte metabolism, and similar mechanisms have also been reported in various horticultural crop species, such as cucumber, tomato, and pepper^154–156^. Furthermore, various studies have shown that drought stress induces the synthesis and accumulation of NO in guard cells, which may also act as a mediator to minimize water loss by participating in ABA-mediated stomatal closure^157^. At the molecular level, NO treatment decreased the levels of drought-induced global DNA methylation of *Dendrobium huoshanense*^158^. Extremely high and low temperatures have deleterious effects on crop growth, development, and yield, particularly at critical phenophases. For example, low temperature is the primary constraint of tomato and pepper (*Capsicum annuum*) yield, since they are typical chilling-sensitive crop species. During the past decade, various studies have demonstrated an accumulation of NO in most cold-stressed crop species, and exogenous NO application triggers cold acclimation and tolerance^159^. Considering the importance of NO-dependent PTMs in NO signaling, increasing amounts of data are available on modifications of *S*-nitrosated proteins following cold exposure in horticultural crop species such as citrus and *Brassica juncea*^160,161^. Given that the identified *S*-nitrosated proteins in cold-stressed plants are mostly involved in metabolism, future studies should focus on the downstream signaling cascades that can be activated by NO-based *S*-nitrosation. Similarly, rapid NO production has also been observed in heat-stressed crops, and NO improves heat tolerance by decreasing ROS contents^162^. High temperature also stimulates NO metabolism, and the total SNO content was shown to increase in heat-stressed pea, citrus, and Brassica^163,164^. With the challenges imposed by global warming, unexpected and increased flood-induced hypoxia will be major agricultural and horticultural production constraints in the near future. During hypoxia, a rapid NO burst has been reported in various plant species, and NO is an essential component to modulate plant acclimation to hypoxic conditions^165^. Overall, by conveying its bioactivity through PTM and interacting with various phytohormones, NO improves plant performance under conditions of nutrient and abiotic stress (Fig. 4).

## NO activates disease resistance in horticultural crops

Pathogens and pests reduce the yield and quality of horticultural crops, causing substantial economic losses and reducing food security across the globe. To survive, crops must sense invading pathogens and mount an effective defense response against these harmful agents. Among these mechanisms are physical barriers such as cell wall thickness and degree of lignification, preformed chemical compounds such as cocktails of diverse secondary metabolites and various transcriptional pathways^166,167^. Insights from decades of plant–pathogen interaction studies have demonstrated that the activation of the signaling network ensures an induced response that is quantitative, timely and coordinated with other activities of the host cells^168^. One of the most prominent generalist messengers is NO, known as the component of the nitrosative burst. In combination with salicylic acid (SA), NO behaves as a crucial component of the plant immune response and participates together with ROS in activating the HR and cell death during incompatible plant–pathogen interactions^9,169^.

NO involvement in the plant immune response was first detected in potato, in which an NO donor induced the accumulation of the potato phytoalexin rishitin, an endogenous antibiotic compound^13^. One year later, it was further proven, following exogenous NO application, that NO could preserve chlorophyll levels in *Phytophthora infestans*-infected potato leaves^170^. Since then, knowledge of the involvement of NO in local and systemic responses against biotic stress has increased greatly. Currently, although some controversy still surrounds the production and turnover of NO in plant immunity, the ensuing early NO burst is thought to orchestrate a plethora of strategies for defense against pathogens. A main strategy is the HR, a localized activation of programmed cell death (PCD) surrounding the infection sites restricting the spread and replication of the pathogens within the plant beyond the initial infection site^171^. *Arabidopsis* infected with *Pseudomonas syringae* pv. tomato DC3000 induced Ca^2+^ influx into the cytosol, activating CaM, which then acts to induce downstream NO synthesis, leading to the HR^172^. Another common feature of pathogen- or elicitor-induced HRs is the rapid accumulation of ROS. At present, a wealth of information supports the simultaneous engagement of nitrosative and oxidative bursts in the plant HR response, and a balance between ROS and NO is required for efficient induction of hypersensitive cell death^173^. For example, both NO and ROS are required for the onset of apoptotic cell death in adjacent cells of oat plants during avirulent crown rust fungal infection^174^. In the cross-talk between pelargonium (*Pelargonium peltatum*) leaves and *Botrytis cinerea*, only an early NO burst concomitant with a periodic increase in H_2_O_2_ concentration was observed in a resistant cultivar^175^. Increasing only one component of the NO–ROS binary system failed to induce hypersensitive cell death in both soybean and tobacco cell suspensions^89,176^, pointing to NO along with ROS as essential components in regulating hypersensitive cell death in pathogen-triggered responses. Moreover, further studies suggested the involvement of NO in cell-to-cell spreading of the HR rather than just in triggering cell death^177^. The immune response is actually mediated by a complex interconnected network that involves various phytohormones and other signaling intermediates. Recent studies have indicated that NO is integrated within this immune network, as it coordinates with several classic pathways, such as SA-related or JA/ethylene-related signaling events^178^.

NO via GSNO-mediated *S-*nitrosation could contribute to well-known immunity signaling pathways and thus has a profound effect on plant immune responses. Immunohistochemical analysis showed that, in sunflower (*H. annuus*) hypocotyls after infection by the fungus *Plasmopara halstedii*, GSNO accumulation was redistributed to epidermal cells, which is the site of penetration by this fungus^179^. The authors suggested that GSNO appears to be a mobile signal and might contribute to sunflower resistance. As mentioned above, GSNOR controls GSNO contents and subsequently global *S*-nitrosation levels. *GSNOR1*-silenced tomato plants presented increased levels of protein *S*-nitrosation, resulting in substantial cell death in response to pathogen infection^180^. It is well known that SA plays important roles in local and systemic responses against pathogen infection, and GSNOR could regulate both the biosynthesis and signaling of SA. In *gsnor1-3 Arabidopsis* mutants, SA-dependent gene expression in response to diminished SA accumulation was compromised, whereas enhanced activity of AtGSNOR1 increased SA-dependent gene expression^60^. Furthermore, in the SA signaling pathway, a large number of proteins undergo *S*-nitrosation^181^. For instance, the *S*-nitrosation of SA-binding protein 3 at Cys 280 suppressed SA-binding capacity and carbonic anhydrase activity^182^, both of which are required for pathogen resistance. It has also been proven that the plant immune response requires conformational changes to NON EXPRESSER OF PATHOGENESIS RELATED 1 (NPR1) via *S*-nitrosation at a specific reactive cysteine, which facilitates the oligomerization of NPR1 and compromises NPR1-mediated disease resistance^68^. Another two potential targets in the SA signaling pathway for *S*-nitrosation are TGA1 and SRG1^183,184^, of which *S*-nitrosation leads to a boost in the immune response. These data illustrate the importance of NO via GSNO-mediated *S-*nitrosation in SA-dependent immune responses.

## NO as a potent fumigant for postharvest control

Numerous studies during the past few years have demonstrated that NO content progressively declines during fruit ripening, with concomitant increases in protein nitration and nitrosation. It has also been suggested that exogenous NO treatment could delay fruit ripening, prevent chilling damage, and affect redox dynamics by improving antioxidant activity and, consequently, increasing nutritional value (Table 1)^185,186^. Here, to avoid repeating, we mainly discussed how NO is a potent fumigant for postharvest control. Postharvest diseases and pests of fruits and vegetables lead to severe losses during handling, transportation, and storage. A large number of studies have shown that NO can maintain the sensory and nutritional attributes and extend the shelf-life of many perishables, including fresh fruits such as strawberry, apple, kiwi, and plum fruits^187–189^, as well as vegetables such as mushrooms, cucumbers, lettuce, and broccoli^190–192^. Increasing evidence shows that NO can act as a fumigant against a wide range of postharvest diseases and pests and has the potential to become an effective and safe synthetic chemical alternative for disease and pest control of fresh produce. It has been demonstrated that NO fumigation is effective for controlling spotted wing Drosophila in strawberry and sweet cherry fruits^193,194^, western flower thrips and aphids in lettuce^195,196^, and codling moths in apple fruis^197^. In recent years, there has been no doubt that NO is effective against all insect and mite species at different life stages, including the egg and pupa stages. It should be noted that small insects such as aphids and thrips in field crops are more sensitive to NO than stored-product insects such as flour beetles. Moreover, NO has obvious inhibitory effects on perishable pathogen-induced diseases. Treatment of cut slices of apple fruits and lettuce with NO prevents surface browning^198,199^. Lai et al. observed that NO donors significantly improved the resistance of apple fruits to *Penicillium expansum*^200^. Similarly, Hu et al. showed that NO inhibited anthracnose (caused by *Colletotrichum gloeosporioides*) in pitaya fruits by activating defense responses and slowing senescence^201^. Supplementation of preharvest green mature tomato fruits with l-arginine, the precursor of NO, induced defenses against postharvest *Botrytis cinerea* diseases in tomato fruits^202^. It should be noted that NO fumigation must be conducted under ultralow oxygen conditions and terminated by N_2_ flushing, avoiding damage to delicate fresh produce caused by nitrogen dioxide, which is spontaneously produced by NO and O_2_. In recent years, NO has also been found to extend the postharvest life of numerous types of flowers^188,203,204^. Although a few studies have pointed to the role of *S*-nitrosation in the potential molecular mechanisms of postharvest disease and pest control, further research is needed to support this conclusion.

NO also diminishes pesticide toxicity and reduces residue in horticultural crops, preventing possible health risks. Pesticide-induced alteration of redox status, resulting from overaccumulation of ROS, negatively affects plant growth and development. As a redox active molecule, NO confers pesticide tolerance to plants in part by mediating antioxidant defense systems^205–208^. In potato plants, NO donors strongly protected against cellular damage induced by diquat and paraquat, two methylviologen herbicides, as a result of the ability of NO to scavenge ROS^206^. In soybean seedlings, NO is able to scavenge ROS induced by the herbicide lactofen and protects plants from oxidative damage. Furthermore, NO application could significantly reduce pesticide residue in plants. This phenomenon could be due to NO-mediated enhancement of some specific enzymes and processes involved in pesticide detoxification, such as the following: hydrolytic enzymes; glutathione; and other conjugation mechanisms, cytochrome P450 oxidases, and peroxidases^209,210^. Yin et al. reported that NO enhanced GSH biosynthesis and promoted the metabolism of chlorothalonil, a widely used fungicide, in tomato seedlings^211^. On this basis, there is no doubt that NO has strong potential for use in horticultural crop protection and could reduce pesticide residue in food crops.

## Conclusions and perspectives

It is now becoming apparent that NO displays myriad physiological and biological functions during the growth, development, environmental interactions, and postharvest storage of horticultural crops. While research on model plant species has provided an abundance of valuable information on horticultural crop species, the potential use of NO in horticulture is still in its infancy, as the application of NO donors is hindered by the instability of these compounds. In this scenario, a promising strategy is the entrapment of NO donors in nanomaterials, which has been successfully applied in the biomedical field. For the first time, Oliveira et al. successfully synthesized NO-releasing chitosan nanoparticles that protected maize plants from salt stress^212^. Therefore, this new technique might have a significant economic impact on both agriculture and horticulture.

*S*-nitrosation is of particular importance for transducing NO bioactivity during plant growth and stress responses. Given that the *S*-nitrosated proteins identified so far are mostly involved in metabolism, future studies should focus on the downstream signaling cascades activated via *S*-nitrosation. On the other hand, stress-induced NO strongly impacts defense-related gene expression, and attention should be paid particularly to the identification and characterization of major transcription factors that are *S*-nitrosated in crop plants.

With the development of sequencing technology, the genomes of some horticultural crop species have been sequenced. Genetic tractability and myriad molecular tools may enable ideal NO-related traits identified in model plant species to be transferred into horticultural species and subsequent production, although genome editing remains challenging.
